# Cardiac Autonomic Neuropathy in Diabetes Mellitus: Pathogenesis, Epidemiology, Diagnosis and Clinical Implications: A Narrative Review

**DOI:** 10.3390/jcm14030671

**Published:** 2025-01-21

**Authors:** Alexandra Gogan, Ovidiu Potre, Vlad-Florian Avram, Minodora Andor, Florina Caruntu, Bogdan Timar

**Affiliations:** 1 Doctoral School of Medicine, “Victor Babes” University of Medicine and Pharmacy, 3000041 Timisoara, Romania; alexandra.gogan@umft.ro; 2 First Department of Internal Medicine, Medical Semiology II, “Victor Babes” University of Medicine and Pharmacy, 3000041 Timisoara, Romania; andor.minodora@umft.ro (M.A.); caruntu.florina@umft.ro (F.C.); 3 Cardiology Clinic, Institute of Cardiovascular Disease, 300310 Timisoara, Romania; 4 First Department of Internal Medicine, Hematology, “Victor Babes” University of Medicine and Pharmacy, 3000041 Timisoara, Romania; 5 Multidisciplinary Research Centre for Malignant Hematological Disease (CCMHM), “Victor Babes” University of Medicine and Pharmacy, 300041 Timisoara, Romania; 6 Department of Diabetes, “Pius Brinzeu” Emergency Hospital, 300723 Timisoara, Romania; avram.vlad@umft.ro (V.-F.A.); bogdan.timar@umft.ro (B.T.); 7 Centre for Molecular Research in Nephrology and Vascular Disease, “Victor Babes” University of Medicine and Pharmacy, 3000041 Timisoara, Romania; 8 Second Department of Internal Medicine, “Victor Babes” University of Medicine and Pharmacy, 3000041 Timisoara, Romania; 9 Multidisciplinary Heart Research Center, “Victor Babes” University of Medicine and Pharmacy, 3000041 Timisoara, Romania; 10 Cardiology Clinic of Timisoara Municipal Clinical Emergency Hospital, 300040 Timisoara, Romania

**Keywords:** cardiac autonomic neuropathy, diabetic neuropathy, cardiovascular risk

## Abstract

**Background**: Cardiac autonomic neuropathy (CAN) is a serious but sometimes underdiagnosed complications of Diabetes Mellitus (DM). Because of the subtle onset and non-specific symptoms that can be mistaken for other conditions, CAN is frequently underdiagnosed despite the serious consequences that can appear. Its significance as an independent risk factor for cardiovascular events, including arrhythmias, sudden cardiac death, and silent myocardial ischemia, is being demonstrated by recent studies. The objective of this review article is to highlight the reasons why CAN is underdiagnosed and its association with decreased cardiovascular risk and promote clinical awareness. This review article summarizes the epidemiology, influence on the cardiovascular system and diagnostic methods of CAN, and the clinical implications of diabetic neuropathy. This review analyzes available data from papers relevant to the topic of diabetic neuropathy, cardiac autonomic neuropathy, and cardiovascular system implications. **Conclusions**: CAN is still underdiagnosed despite its clinical impact because routine screening is lacking, and healthcare providers are not aware of it. To improve outcomes for people with DM, it is necessary to introduce standardized diagnostic procedures into clinical practice and increase the knowledge about CAN.

## 1. Introduction

Diabetes mellitus (DM) is one of the most prevalent chronic diseases around the world, making it a significant public health concern [1]. Worldwide, the prevalence of diabetes is high and is continually increasing. Numerous variables could be responsible for an increase in the prevalence of diabetes at the population level. Although obesity is frequently cited as the primary cause of the rising incidence of diabetes, other variables have also been found to have potential significance, including ethnic background, lifestyle, socioeconomic level, education, and urbanization. Other research indicates that various population-level factors influence rising diabetes incidence rates including environmental pollution, an environment that promotes obesity, and rapid socioeconomic development [2]. Other factors, such as improved case detection, lower mortality, and a real increase in incidence, may also contribute to the rise in prevalence in this condition [3]. It was anticipated that 10.5% of people between the ages of 20 and 79 had diabetes in 2021, and that number would rise to 12.2% in 2045. The prevalence of diabetes was highest among people aged 75–79 years, and it was very similar among men and women. According to estimates, the prevalence was higher in nations with high incomes (11.1%) than in low-income countries (5.5%), and in urban regions (12.1%) than in rural ones (8.3%) [4]. This epidemic is particularly focused on type 2 diabetes (T2D), which is responsible for approximately 90% of all cases of diabetes worldwide. The increasing prevalence of T2D has been attributed to several causes, including an aging population and an increase in obesity in industrialized nations [5]. Diabetes is a complicated, demanding condition that can contribute to severe morbidity, mortality, and a deterioration in quality of life due to the complications that appear over time [1]. The burden of diabetic neuropathies is also increasing [5]. Diabetic neuropathy develops in at least 50% of people with diabetes over time, and the length of the disease also affects the prevalence of diabetic neuropathy. In fact, after ten years of follow-up, the percentage of individuals with T2D who had diabetic neuropathy increased from 8 to 42% [6]. Most diabetic neuropathies are produced by hyperglycemic effects on small and large fiber nerves, and glycemic management in people with T1D reduces the occurrence of neuropathy. Other factors, especially those related to the metabolic syndrome, are important in individuals with T2D and should be addressed. Although distal symmetric polyneuropathy (DSPN) is the most common type of neuropathy, autonomic syndromes, particularly cardiovascular autonomic neuropathy (CAN), have a higher mortality rate. Identifying and accurately diagnosing diabetic neuropathies is critical to limiting progression. Until improved disease-modifying treatments are developed, management will remain focused on diabetes, metabolic risk factor control, and complications management [7].

## 2. Diabetic Neuropathy

Diabetic neuropathy is the most prevalent chronic complication of diabetes and is represented by a heterogeneous set of clinical or subclinical manifestations affecting the peripheral nervous system (PNS) [8,9]. The Americans with Disabilities Act (ADA) defines diabetic neuropathy as “the detection of peripheral nerve dysfunction symptoms and/or signs in individuals with DM after other causes have been ruled out” [10]. Diabetic neuropathy risk is proportional to the length and severity of hyperglycemia, as is the case with other microvascular issues. After 25 years, half of patients developed neuropathy, and the onset of neuropathy was strongly associated with the length of DM [11]. There are various clinical syndromes for diabetic neuropathy, such as distal symmetric polyneuropathy (DSPN), autonomic neuropathy, and thoracic and lumbar nerve root disease. This results in polyradiculopathies, focal mononeuropathies caused by individual, cranial and peripheral nerve involvement, particularly affecting the median and oculomotor nerves, and asymmetric involvement of multiple peripheral nerves, which results in a mononeuropathy multiplex [8].

### 2.1. Distal Symmetric Polyneuropathy

The most prevalent kind of diabetic neuropathy is DSPN [12]. After 20 years of being diagnosed with T1D, a minimum of 20% of patients may develop DSPN, according to the DCCT/EDIC [5,13]. At least 10 to 15% of patients with T2D who are newly diagnosed may have DSPN, and after ten years, the prevalence rises to 50% [14,15]. Significant associations between the development of diabetic peripheral neuropathy and factors such as age, length of diabetes disease, height, HbA1c, smoking, proliferative or background diabetic retinopathy, high-density lipoprotein cholesterol, and cardiovascular disease were found throughout the EURODIAB IDDM Complications Study [16]. Pain and dysesthesia are the most prevalent early symptoms brought on by small fiber involvement. Up to 25% of people with DSPN have neuropathic pain, which may be the initial symptom that leads them to seek medical attention. The pain is characterized by tingling, burning, lancinating, or shooting and usually gets worse at night. Allodynia, or pain triggered by contact, and hyperalgesia, or an excessive reaction to painful stimuli, are two conditions that can accompany neuropathic pain. A decrease in health-related quality of life, physical or psychosocial impairment, and disruption of everyday activities can all result from neuropathic pain [8]. Large fiber involvement can result in loss of protective feeling, tingling with no pain, and numbness. A potential risk factor for foot ulcers caused by diabetes is the loss of protective feeling, which is a sign of DSPN [17]. Sensory loss to temperature, vibration, and gentle touch is revealed by physical examination. Abnormalities in many peripheral sensory tests are more than 87% sensitive in identifying neuropathy. There is insufficient evidence to support lifestyle modification or glycemic control as treatments for neuropathic pain in patients with diabetes or prediabetes, leaving mainly pharmacological approaches [8]. The European Medicines Agency, Health Canada, and the U.S. FDA (Food and Drug Administration) have authorized pregabalin and duloxetine for the medical management of neuropathic pain in individuals with DM [18].

### 2.2. Focal and Multifocal Neuropathies

Patients with diabetes are more likely than those without the disease to develop mononeuropathies. They may arise from the involvement of the common peroneal, radial, ulnar and median nerves. Rare and acute, cranial neuropathies mostly affect the III, IV, VI and VII cranial nerves and typically go away on their own over a period of months [8]. Usually affecting the lumbosacral plexus, diabetic radiculoplexus neuropathy is also known as diabetic amyotrophy or diabetic polyradiculoneuropathy. Extreme unilateral thigh pain, weight loss, and motor weakness are common symptoms of the illness. Men with T2D are more likely to experience the problem. To record the extent of the disease and other etiologies, such as infectious, and inflammatory spinal disease, degenerative disc disease or neoplastic, electrophysiological evaluation is necessary [19].

## 3. Diabetic Autonomic Neuropathy

**Figure 1:**
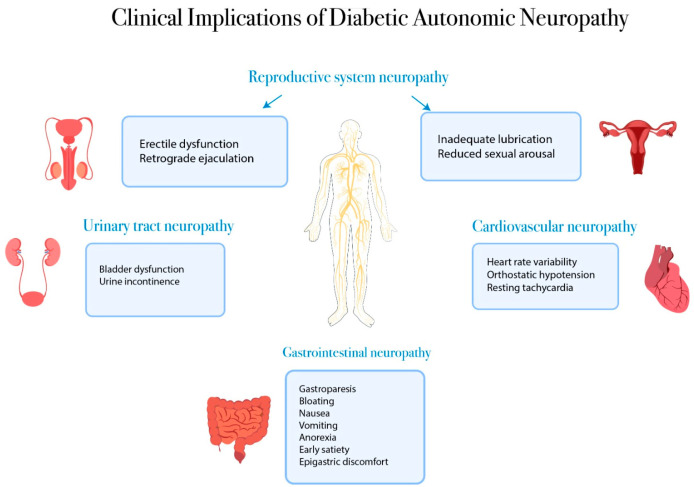
Clinical implications of diabetic autonomic neuropathy in T1D and T2D.

One type of neuropathy that is frequently seen in individuals with DM is diabetic autonomic neuropathy (DAN). The longer a person has diabetes, the higher the prevalence of DAN. Up to 7% of people may have DAN at the time of their diabetes diagnosis, but after 15 years, it may reach 50% [20,21]. Patients with either T2D or T1D experience DAN [22]. Figure 1 describes the clinical implications of DAN.

### 3.1. Gastrointestinal Neuropathy

Any part of the gastrointestinal system may be affected by gastrointestinal neuropathies, which can present as constipation, diarrhea, fecal incontinence, esophageal dysmotility, and gastroparesis [23]. T1D had a higher overall prevalence of gastroparesis over a ten-year period (5%) than patients with T2D (1%) and control participants (1%), according to the only community-based investigation [23]. The mismatch between the absorption of food and the pharmacokinetic characteristics of insulin and other drugs may result in gastroparesis, which can have a direct impact on glycemic control and cause glucose fluctuation and unexplained hypoglycemia. Most patients with gastroparesis have had diabetes for a long time [23]. Most diabetic gastroparesis is clinically asymptomatic, but severe diabetic gastroparesis represents one of the most crippling of all diabetic gastrointestinal problems. This disorder’s main clinical manifestations are bloating, nausea, vomiting, anorexia, early satiety, and epigastric discomfort. Vomiting or nausea episodes can occur in cycles or last anywhere from a few days to several months [24]. Crucially, it is well known that severe blood glucose fluctuations, hyperglycemia, and hypoglycemia, as well as some drugs, particularly opioids, other painkillers, and glucagon-like peptide 1 receptor agonists, can modify gastric emptying. Therefore, before a definitive diagnosis is made, all these factors that are known to affect stomach emptying should be considered. The measurement of stomach emptying by scintigraphy of digestible solids at 15-min intervals for 4 h after food consumption is the gold standard for diagnosis. However, the 13C-octanoic acid breath test is becoming a competitive substitute [23]. The FDA has only approved metoclopramide as a therapy for gastroparesis [22]. However, there is not much evidence to support the use of this medication for the treatment of gastroparesis, and because of the possibility of severe side effects, it is no longer advised to use this medication for longer than five days [23].

### 3.2. Genitourinary Neuropathy

In both men and women, lower urinary tract symptoms, such as bladder dysfunction and urinary incontinence, are associated with diabetic neuropathy [19]. Urinary tract infections, particularly pyelonephritis, are more likely to occur in people with bladder dysfunction, which may induce or worsen renal failure [23]. Retrograde ejaculation and/or erectile dysfunction (ED) are two symptoms of diabetic autonomic neuropathy in males. Men with diabetes are three times more likely compared to those without the condition to experience erectile dysfunction [25]. Women with diabetes are susceptible to reduced sexual arousal and insufficient lubrication, and they may experience increased pain and decreased sexual desire during sexual activity [23].

### 3.3. Cardiac Autonomic Neuropathy

Cardiac autonomic neuropathy (CAN) is arguably among the most frequently disregarded of all the major consequences of diabetes and leads to the cardiovascular autonomic nerve system dysfunction (EDIC) [26,27]. The impairment of cardiovascular autonomic function in individuals with confirmed DM after alternative reasons have been ruled out is known as CAN, according to the Toronto Consensus Panel on Diabetic Neuropathy Subcommittee [28]. About 7% of people with T1D or T2D had CAN identified at diagnosis [29]. For people with DM type 1 and type 2, the risk is thought to rise by roughly 6% and 2% every year, respectively [20]. The prevalence of CAN rises significantly with the length of diabetes, even if it is relatively low in newly diagnosed individuals with T1D. In the DCCT/EDIC cohort, incidence rates of at least 30% were noted over twenty years of diabetes duration. In patients who are diagnosed with T2D, the incidence of CAN also rises with the length of disease and may reach 60% of patients after 15 years [29]. There is disagreement on how gender affects CAN. There were no variations in the frequency of CAN between men (35%) and women (37%), according to the EURODIAB IDDM Complications Study, but according to the ACCORD study, women (4.7%) were more likely than males (2.6%) to have CAN [30,31]. Autonomic neuropathy was significantly correlated with the duration of diabetes, age, glycated hemoglobin, retinopathy, microalbuminuria, hypoglycemia, ketoacidosis, smoking, low HDL cholesterol, total cholesterol/HDL cholesterol ratio, fasting triglycerides, and diastolic blood pressure. According to the EURODIAB IDDM Complications Study, autonomic neuropathy was linked to a higher risk of cardiovascular disease [32]. Based on the results of multiple epidemiological studies conducted among people with diabetes, those with CAN have a five-fold greater 5-year mortality rate from this significant consequence than people without cardiovascular autonomic dysfunction [33]. Numerous processes and pathways contribute to CAN, which is brought on by intricate interconnections and ultimately results in neuronal ischemia and death. Hyperglycemia is the primary cause of the pathogenic process. Oxidative stress brought on by hyperglycemia and harmful advanced glycosylation products alter endothelium, membrane permeability, and mitochondrial functioning. Several cellular processes, communication between cells and the surrounding matrix, transcription factors, and gene expression are all disrupted by these various routes. All of this results in neuronal death and malfunction [25]. A variety of clinical presentations, including subtle appearances of symptoms at different stages of its natural history, are what define CAN. Nerve length has an inverse association with the progression of nervous system injury, with the vagus nerve being impacted first. Sympathetic predominance results from parasympathetic system failure in early illness. Clinically, this manifests as tachycardia at rest. A failure to react appropriately to physiological stresses, such as exercise, is a result of advanced CAN, which causes a constant heart rate. Exercise intolerance is the clinical manifestation of this reduced response [34].

## 4. Cardiovascular Implications of CAN

When patients with DM have simultaneous coronary artery disease (CAD), silent myocardial ischemia (SMI) is the most common clinical sign of CAN. CAN was a powerful predictor of SMI and later cardiovascular events at T2D patients included in the Detection of Ischemia in Asymptomatic Diabetics (DIAD) trial [35]. Vinik et al. found a consistent correlation between CAN and the occurrence of SMI as determined by exercise stress testing in a systematic review and the prevalence rate ratios ranged from 0.85 to 15.53 [20]. Having T2D increases the risk of sudden cardiac death (SCD) two- to fourfold, especially after a myocardial infarction (MI). The most common pathogenic substrate for adult SCD is CAD. The most prevalent electrophysiological mechanism is ventricular fibrillation, and a major contributing factor to the onset of a cardiac arrest is thought to be compromised cardiac autonomic regulation. Heart rate recovery post-exercise, heart rate instability, deceleration ability, baroreflex sensitivity, and HRV were all independent indicators of cardiac death following myocardial infarction, although their predictive value was higher when combined. None of them, however, have been shown to be able to accurately identify patients who would benefit from preventive treatment with an implanted cardioverter defibrillator [36]. Both sudden death and a high incidence of cardiac arrhythmias are linked to CAN. A fatal arrhythmia may eventually be caused by significant but asymptomatic ischemia, which could be one reason for SCD. The autonomic nervous system’s sympathetic and parasympathetic components are out of balance, which directly explains deadly arrhythmias. Malignant ventricular arrhythmias can also result from other autonomic imbalance-related causes, such as decreased awareness of hypoglycemia, persistent hypoglycemia episodes, and poor response to hypoxic states, which can ultimately cause sudden death [37]. QT prolongation brought on by an autonomic imbalance may also put people at risk for sudden death and potentially fatal cardiac arrhythmias [37]. Male patients with decreased heart rate variability (HRV) had a larger corrected QT prolongation than male patients without this complication, according to the EURODIAB IDDM Complications Study results [38]. Coronary artery calcification was also linked to autonomic dysfunction, which was measured by decreased heart rate variability. MRI imaging revealed that CAN is linked to higher mass of the LV and concentric remodeling. Left ventricular dysfunction, however, may also be caused by anomalies other than CAN in DM patients, which includes interstitial myocardial fibrosis, microangiopathic or metabolic alterations. However, the strong sympathetic tone caused by the parasympathetic denervation seen in the early stages of CAN promotes metabolic alterations, such as elevated cardiac catecholamine levels [37]. Atherosclerosis has been linked to alterations in the phenotypic of vascular smooth muscle cells caused by sympathetic denervation, including dedifferentiation, migration to the intima, and formation of extracellular matrix [37].

## 5. Implications of CAN

Even though CAN is subclinical, exacerbations and remissions are typically characteristic features of symptoms when they do manifest. Some patients’ symptoms do not get worse with time, while others’ symptoms stay constant [39]. In the early phases of autonomic dysfunction, anomalies are recorded as a drop in heart rate variability (HRV) long before alterations in resting heart rate are noticeable. Afterwards, resting tachycardia develops when the parasympathetic branch of the autonomic nervous system is impacted. Heart rate adaptation to exercise, stress, and sleep is impaired after approximately five years of delay, when sympathetic cardiac denervation begins. The heart rate returns to normal, but it is still higher than in healthy people. In more severe phases, total heart denervation causes the heart rate to become fixed and unresponsive [39]. A stable heart rate which is not affected by mild stress, exercise, or sleep is a sign of severe CAN and nearly total cardiac denervation [40].

Orthostatic hypotension (OH) in individuals with DM is typically caused by injury to the efferent sympathetic vascular fibers, especially in the splanchnic vasculature. OH is diagnosed when systolic blood pressure drops by more than 20 mmHg, or diastolic blood pressure drops by more than 10 mmHg [40]. Symptoms of presyncope and dizziness are common in patients with orthostatic hypotension. Orthostatic hypotension may also be the cause of symptoms like neck pain, fatigue, dizziness, and blurred vision. However, even after their blood pressure has significantly decreased, many people continue to have no symptoms [8]. There can be a lower norepinephrine response in people with orthostatic hypotension in comparison to the drop in blood pressure. A decrease in blood volume or decreased red cell mass may also be significant, as can decreased cardiac output and acceleration. Orthostatic symptoms may be made worse by other variables such postprandial blood accumulation, insulin’s hypotensive effect, and the use of diuretics to treat renal or heart failure, which causes volume depletion [41].

Tolerance for exercise can be reduced by autonomic dysfunction. It has also been demonstrated that there is an inverse relationship between the degree of severity of CAN and either the maximal elevated heart rate or any rise in heart rate during exercise. Additionally, exercise tolerance is limited by systolic dysfunction, diastolic filling, and reduced ejection fraction [42]. It is recommended that individuals with DM at risk for CAN undergo cardiac stress testing before starting an activity regimen [43].

Individuals with DM had two to three times the perioperative cardiovascular morbidity and mortality rate of individuals without DM [44]. According to Burgos et al., individuals with DM with autonomic dysfunction need vasopressor treatment more frequently than those without. The vasodilatory effects of anesthesia were not entirely offset by the typical autonomic reaction of tachycardia and vasoconstriction [45]. CAN has also been linked to more severe intraoperative hypothermia, according to recent research by Kitamura et al. [46]. Reduced hypoxic-induced ventilatory drive requires preoperative CAN [47].

## 6. CAN Diagnosis

Cardiovascular reflex tests, heart rate variability spectrum analysis, baroreflex sensitivity, muscle sympathetic nerve activity, catecholamine evaluation, cardiovascular sympathetic tests, and heart sympathetic imaging are some of the clinically used CAN assessment techniques. Table 1 summarizes the various tests and the positive diagnosis for CAN. Also, Table 2 summarizes the advantages and disadvantages of CAN diagnostic methods.

### 6.1. Cardiovascular Reflex Tests (Ewing Tests)

Five fundamental tests for noninvasive autonomic examination were reported by Ewing et al. in the early 1970s, such as the Valsalva maneuver, the heart rate response to standing, the heart rate response to breathing, the blood pressure response to standing, and the blood pressure accordance to sustained handgrip [53]. The Toronto Consensus Panel’s CAN Subcommittee claims that a single abnormal test is adequate to identify potential or early CAN. Predictable CAN is indicated by more than one abnormal test. Severe CAN is indicated by orthostatic low blood pressure in addition to aberrant test results [54].

Using a supine position, the deep breathing test (DB) measures heart rate variability from beat to beat. The parasympathetic nervous system primarily controls the response of the heart to deep breathing, while the sympathetic nervous system’s activity may have an impact on this measurement [55]. During electrocardiogram (EKG) recording, a metronome or similar device paces the patient’s breathing at six breaths per minute. The ratio of a respiration cycle’s three maximum and three minimum respiration rate intervals is known as the expiration-to-inspiration (E/I) index. E/I ratios below the threshold and variations in HR <10 bpm are regarded as abnormal [55].

To assess the response of the heart rate (HR) to the Valsalva maneuver, a patient is requested to forcefully exhale for 15 s to 40 mmHg on a manometer, while an EKG is being recorded concurrently. Bradycardia develops following release, and tachycardia occurs during exhale in healthy individuals. Calculated as the ratio of the shortest RR interval through exhalation to the longest RR duration following expiration, the norm should be 1.2 or above [55]. This produces a four-phase reflex response with approximately 80% specificity that is controlled by both the sympathetic and parasympathetic autonomic nervous systems [48].

The patient transitions from a supine to a standing posture during the Lying-to-Standing (LS) test, requiring continuous EKG monitoring. Atropine produces a parasympathetic blockade that significantly reduces the normal heart rate while standing. The typical and quick rise in heart rate that occurs in healthy persons in reaction to standing peaks about the fifteenth beat after standing [20]. After changing position, the shortest RR interval between the tenth and twentieth beat and the largest RR interval between the twenty-fifth and thirty-fifth beat are measured. Ratios of 30:15 and values less than 1.03 are deemed abnormal [55].

The response of blood pressure (BP) to standing is first assessed while supine and then two minutes after standing up. Baroreflex-mediated vasoconstriction of the peripheral vessels and tachycardia quickly rectify the blood pressure drop caused by the instantaneous collecting blood in the dependent circulation in healthy persons [20]. An inappropriate or borderline blood pressure response is indicated by a drop in blood pressure of more than 30 mmHg or between 10 and 29 mmHg. Additionally, orthostatic incompetence is also indicated by a drop in blood pressure of more than 20 mmHg in the systolic or more than 10 mmHg in the diastolic [55].

Using a handgrip dynamometer, the test measures the diastolic blood pressure response to sustained handgrip, which is the increase in heart rate and systolic and diastolic blood pressure caused by prolonged muscle contraction. The dynamometer is held at 30% maximum for five minutes after being squeezed to isometric maximum. A rise in diastolic blood pressure of more than 16 mmHg is considered normal, whereas a rise of less than 10 mmHg is deemed problematic [20]. This exam assesses the sympathetic nervous system [39].

### 6.2. Spectral Analysis of Heart Rate Variability

Increased heart rate variability (HRV) in healthy individuals is seen as an indicator of autonomic integrity, while decreased heart rate variation is a precursor to autonomic dysfunction. HRV analysis can be done in the frequency and time domains for brief intervals of a few minutes (<10 min) or for 24 h records. The examination of average normal to normal interval, maximum heart rate difference, average heart rate, daytime and nighttime heart rate variation, R-R interval standard deviation, root-mean-square of the variation of subsequent R-R intervals, 5 min average of normal R-R interval standard deviation, and the number of times per hour that two successive R-R intervals differ by over 50 ms are all included in time domain analysis [29]. After data transformation, such as the rapid Fourier transform, to produce HRV spectra, frequency-domain analysis is carried out. These histograms show the effects of both parasympathetic and sympathetic branches on HR and are separated into low frequency (LF) (0.04–0.15 Hz) and raised frequency (HF) (0.15–0.4 Hz) regions. An indicator of autonomic balance, the sympathetic-to-parasympathetic ratio (LF/HF) is the primary usage for this ratio. In early CAN, a decrease in the HF band could suggest vagal dysfunction. However, simultaneous instability of sympathetic activity may be indicated by a decrease in the LF band [48,56]. Spectral analysis is also more patient-friendly, more sensitive in the early phases of CAN, and does not require excellent collaboration [57]. Ziegler et al. [58] used 21 patients with DM and 120 healthy volunteers to compare their own tests. Low, mid, and high frequencies were classified as 0.01–0.05 Hz, 0.05–0.15, and 0.15–0.5 Hz, respectively, for power spectral analysis testing conducted on participants who were at rest. The typical Ewing series of tests, which included time-domain measurements such as the 30:15 ratio, max-min, E:I ratio, Valsalva ratio, and coefficient of variation, was used as well in this investigation. The findings of HRV tests showed that associations between time-domain measurements were best found in the high-frequency band and the outcomes of most tests had a significant correlation with each other, except for the Valsalva ratio [59].

### 6.3. Spontaneous Baroreflex Sensitivity

Short-term blood pressure is maintained by the arterial baroreceptor reflex. Two main effector pathways are triggered by activating the baroreceptors. While sympathetic vasoconstrictor activity suppression lowers peripheral vascular resistance, cardiac vagal fiber stimulation decreases heart rhythm and, thus, increases cardiac flow. The stimulation of baroreceptors continuously activates a variety of reflex mechanisms that maintain the vagal tone. As a result, the responsiveness of the baroreceptors determines the amount of the vagal tonus. The myocardial muscle’s electrical stability is preserved by the cardioprotective effects of the vagus nerve. Therefore, a decrease in baroreceptor sensitivity is a risk factor for cardiovascular disease [58,60].

The diagnostic range for patients with diabetes having CAN has been extended by the creation of a servoplethysmomanometry-based method and it is computed by measuring the heart rate-blood pressure relationship following an intravenous phenylephrine bolus [26,61]. Finapress, a technique that enables the evaluation of neural regulation of the sinus node’s activity using arterial baroreceptors, is increasingly being used to test the operation of the autonomic nervous system. In response to an abrupt rise in blood pressure, it assesses the capacity to reflexively raise vagal activity and lower sympathetic activity. Research protocols use it to evaluate sympathetic baroreflex and cardiovagal function [26]. In major worldwide multicenter prospective research with patients diagnosed with DM and a recent myocardial infarction, the Autonomic Tone and Reflexes After Myocardial Infarction (ATRAMI) study, the baroreflex sensitivity (BRS) was a substantial independent risk factor of cardiac mortality [26].

### 6.4. Imaging Techniques

With positron emission tomography (PET) with either [123I]-meta-iodobenzylguanidine (MIBG) or [11C]-meta-hydroxy-ephedrine ([11C] HED), a quantitative scintigraphic evaluation of the sympathetic innervation of the human heart is feasible [62,63]. Norepinephrine absorption in postganglionic sympathetic neurons is facilitated by the nonmetabolized norepinephrine analogue MIBG. Autonomic reflex tests have shown in multiple investigations that patients with CAN have reduced myocardial MIBG absorption [42]. While there is no retention of MIBG among patients with advanced disease, the posterior and inferior sections of the left ventricle exhibit a predominance of decreased MIBG uptake [64]. Defects in cardiac innervation have also been studied using hydroxyephedrine (HED), an analogue of norepinephrine [11C]. Proximal hyperinnervation and terminal hypoinnervation are seen in CAN individuals with DM after [11C]-HED imaging. Potentially fatal cardiac electrical instability could arise from this kind of proximal hyperinnervation in comparison to the distal denervation. Due to the correlation between myocardial dysinnervation and myocardial blood flow control deficits, such as a reduction in blood reserve, it has been proposed that myocardial damage in diabetes may be caused by both poor myocardial perfusion and increased cardiac sympathetic tone [64].

### 6.5. Microneurography

The only way to measure sympathetic activation directly and continuously is to measure muscle activity in sympathetic nerves using an invasive technique. It is possible to capture spikes in electrical activity in the radial, tibial, or peroneal muscle nerves. While individuals with Type 1 diabetes experience fewer sympathetic bursts, individuals with Type 2 diabetes have been shown to exhibit higher muscle activity in their sympathetic nerves, likely linked to autonomic dysfunction. This technique’s employment is restricted due to its intrusive nature and its high workforce, time, and interpretation requirements [39].

### 6.6. Catecholamine Asessment

Catecholamines and their precursors or metabolites, such as noradrenaline, adrenaline, 3,4-dihydroxy-l-phenylalanine and 3,4-dihydroxyphenylglycol, are measured in plasma to reflect systemic autonomic activation [65]. However, measuring catecholamine plasma concentrations for the purpose of identifying CAN in diabetes patients has a limited diagnostic power [42,66].

## 7. Conclusions

Nonspecific symptoms and insidious onset of CAN lead to underdiagnosis, and therefore patients may become vulnerable to complications, such as silent myocardial ischemia, arrhythmias, and sudden cardiac death. Nevertheless, the screening efforts for CAN remain inadequate in clinical practice. A multitude of clinical studies have provided direct information about CAN as an underdiagnosed complication of diabetes. Therefore, in the EURODIAB IDDM Complications Study [30], the prevalence of CAN in patients diagnosed with T1D is increasing, but remains mostly undiagnosed, due to non-specific symptoms and the lack of routine tests to evaluate cardiovascular autonomic function. Also, in the Diabetes Control and Complications Trial (DCCT) [67] and the Epidemiology of Diabetes Interventions and (EDIC) [68], it was mentioned that CAN, in the early stages, may have absent symptoms, and CAN can progress without being noticed.

### Gaps in Evidence

As the highest prevalence of DM is represented by patients who have diabetes for a long time and it has an independent role in exacerbating cardiovascular mortality and morbidity, there is an urgent need to implement standardized diagnostic tests. Finally, the integration of regular screening protocols and CAN awareness in the medical field are essential in the therapeutic and diagnostic gaps. Therefore, early diagnosis can be made, and cardiovascular risk can be reduced in at-risk populations.

## Figures and Tables

**Table 1:** Principles methods and positive diagnosis of CAN.

Methods of Diagnosis	Principles of the Methods	Positive Diagnosis
Cardiovascular Reflex Tests (Ewing Test)	Heart rate response to Valsalva maneuver The patient lies supine, exhales forcefully through an open glottis on a manometer that produces 40 mmHg of pressure for 15 s and then resumes regular breathing. Both the sympathetic and parasympathetic autonomic systems mediate the ensuing four-phase reflex response.	When the longest to shortest R-R interval ratio is less than 1.2, it is deemed abnormal.
	Heart rate response to standing The parasympathetic influence on cardiovascular measures following a postural shift from a supine to a standing position is assessed by this test. In healthy individuals, there is a sharp rise in heart rate that peaks around the 15th systole, then followed by relative bradycardia that peaks around the 30th systole.	When the R-R interval ratio that exists between the 30th and 15th systoles is less than 1.03, it can be considered abnormal.
	Systolic blood pressure response to standing The patient is placed in the supine posture for this examination. The patient quickly stands up to stand after a brief interval of rest. Both at rest and two minutes after rising, the systolic blood pressure is recorded.	A fall of less than 10 mmHg is considered normal, a fall between 10 and 29 mmHg is considered borderline, and a drop of more than 30 mmHg accompanying symptoms is considered abnormal.
	Diastolic blood pressure response to isometric exercise In order to assess the sympathetic nervous system, the patient must do a handgrip with the greatest amount of effort possible. Currently, this diagnostic technique is not regarded as a standard test.	A rise in diastolic blood pressure of greater than 16 mmHg in the contralateral arm is the usual reaction; a response of less than 10 mmHg is deemed abnormal.
	Beat-to-beat HRV Clinical CAN in its early stages has been linked to a decrease in HRV. In healthy individuals, both parasympathetic and sympathetic activity mostly influences the beat-to-beat fluctuation with respiration. The patient lies supine and at rest, and their heart rate is tracked by an ECG as they breathe in and out at a rate of six breaths per minute.	It is considered normal when the heart rate differs by more than 15 beats per minute, and pathological when it is less than 10 beats per minute.
Spectral Analysis Of Heart Rate Variability	This is a mathematical formula that breaks down HRV into its causative elements and displays them based on how frequently they modify the RR. A graph with amplitude vs. frequency displays the spectral amplitude. The HRV magnitude and oscillations at various frequencies, or the number of HR changes per second, are both reflected in the spectrum amplitude.	Very low-frequency component, or VLF (between 0.01 and 0.04 Hz): this element is linked to vasomotor tone variations linked to sweating and thermoregulation (sympathetic control); The low-frequency component, or LF, which ranges from 0.04 to 0.15 Hz, is associated with the baroreflex, which is vagal modulation and sympathetic regulation; The high-frequency component, or HF (between 0.15 and 0.5 Hz), is associated with variations in RR based on the parasympathetic control of the breathing phases.
Spontaneous Barorefelex Sensitivity	This technique calculates how variations in systolic blood pressure affect HR. Pharmacological procedures (intravenous bolus administration of epinephrine) or non-pharmacological techniques (physical maneuvers such postural alteration) can be used to complete the test. Both methods require a continuous blood pressure measurement as well as a continuous, synchronized HR (R-R interval) measurement.	In the heart and blood arteries, the baroreflex mechanism controls both sympathetic and vagal fluxes. Reduced BRS is a sign of impaired vagal responses, which can lead to prolonged adrenergic activity.
Imaging Techniques (Pet And Spect)	In the context of different disorders, radionuclide imaging methods can be utilized to measure the heart’s sympathetic innervation. Newer radiotracers that bind to post-synaptic alpha and beta receptors are also being devised and studied, although the majority of radiotracers that have been developed image pre-synaptic anatomy and function. Although several noninvasive tests are useful in identifying diabetic-induced neuropathy, they mostly evaluate parasympathetic function. Assessing sympathetic function using cardiac imaging may offer a special method for evaluating neuropathy.	In 40% of patients with DM without autonomic neuropathy, Stevens and colleagues discovered anomalies of 11C-HED retention, which initially manifested in the inferior wall before moving to other areas of the heart. Increased absolute tracer retention (hyperinnervation) in proximal myocardial segments along with reduced retention (denervation) in distal segments were observed in patients with severe neuropathies. This innervation pattern may lead to electrical instability and put patients at risk for potentially fatal arrhythmias.
Microneurography	Microelectrodes placed into a fascicle of a distal sympathetic nerve to the skin or muscle (microneurography), more frequently at the level of the peroneal nerve, can be used to directly record and measure spikes of efferent sympathetic activity in the skeletal muscle at rest or in response to different physiological perturbations. The frequency of muscle sympathetic nerve activity bursts rises when blood pressure drops and vice versa, and they are linked to the inhibitory action of systole on arterial baroreceptors.	Patients with type 2 diabetes who have noradrenergic autonomic dysfunction have been found to have increased resting muscle sympathetic nerve activity and decreased reactivity to physiological hyperinsulinemia or glucose consumption, which is similar to insulin-resistant states and obesity. The total amount of bursts is significantly reduced, by around half, in people with type 1 diabetes [26,48,49,50,51,52].

**Table 2:** Advantages and disadvantages of CAN diagnostic methods [26,48,52].

Methods of Diagnosis	Advantages	Disadvantages
Cardiovascular Reflex Tests (Ewing Tests)	They are extensively used and standardized. The tests do not entail intrusive procedures or patient danger. They are sensitive in early identification, even before symptoms develop. They examine many aspects of the autonomous function.	External influences like drugs, stress, and exhaustion can impair the accuracy of the tests. The sensitivity of the test can vary, and the findings can differ based on the method utilized.
Spectral Analysis Of Heart Rate Variability	Along with CARTs, HRV analysis is a clinically meaningful metric that offers important insights into the cardiovascular system’s autonomic control. HRV can be analyzed with statistical indices in both the frequency and time frame domains.	A strategy for spectrum decomposition is absent while applying autoregressive approach. The power spectrum is misinterpreted because of irregular breathing patterns and verbalization, which produce incorrect “sympathetic overactivity” and artifactual low frequencies.
Spontaneous Barorefelex Sensitivity	The spontaneous BRS process is quick and easy to use. It is a specialized beat-to-beat non-intrusive blood pressure monitor that is necessary for all BRS procedures. Because it integrates data from blood pressure and heart rate, cardiac vagal BRS evaluation is a crucial part of autonomic testing. One well-known independent predictive measure for cardiovascular morbidity and mortality in the general population is cardiac vagal BRS.	Variations brought on by the harmless blood pressure monitors’ drifts; age-associated decline in BRS. Slow breathing raises BRS and lowers sympathetic efferent drive, even though BRS measurements often do not require rigorous respiratory pattern control.
Imaging Techniques (Pet And Spect)	The structural viability of the sympathetic nerve system’s supply to the heart is directly evaluated using scintigraphic tracers. Most secondary care facilities have single photon emission computed tomography and [123I]-MIBG scanning available and in widespread usage. Although scintigraphic data is more sensitive in identifying alterations in sympathetic neural anatomy and/or function, it corresponds with HRV testing.	There are no widely accessible parasympathetic tracers presently. There is no conventional methodology for evaluating sympathetic integrity. No standards values have been established. Myocardial perfusion has a significant impact on tracer delivery. The body mass index, diastolic blood pressure, and local variables that affect tracer absorption and retention all have an impact on [123I]-MIBG retention. Both 6-[18F]-dopamine positron emission tomography and [11C]-HED are not reimbursed and are not widely available.
Microneurography	It is the only technique that makes it possible to assess sympathetic nerve conduction directly and continuously.	The procedure’s time-consuming nature and invasiveness. It is not recommended for regular autonomic evaluation. It needs a skilled operator with knowledge and cannot be repeated frequently in the same subject.
Catecholamine Asessment	The causes and mechanisms of CAN has been better understood. Concentrations of plasma catecholamines can reveal the activity of the adrenomedullary and sympathetic noradrenergic hormone systems.	The concentration of norepinephrine in plasma rises with age. Smoking raises catecholamine levels and sympathetic nerve activity. For comparisons, a 24 h smoke abstention period is necessary. Because position, stress, and the surrounding temperature all have an impact on catecholamine concentrations, they need to be controlled.
